# The Motor Protein KIF14 Inhibits Tumor Growth and Cancer Metastasis in Lung Adenocarcinoma

**DOI:** 10.1371/journal.pone.0061664

**Published:** 2013-04-23

**Authors:** Pei-Fang Hung, Tse-Ming Hong, Yi-Chiung Hsu, Hsuan-Yu Chen, Yih-Leong Chang, Chen-Tu Wu, Gee-Chen Chang, Yuh-Shan Jou, Szu-Hua Pan, Pan-Chyr Yang

**Affiliations:** 1 Graduate Institute of Life Sciences, National Defense Medical Center, Taipei, Taiwan; 2 Institute of Clinical Medicine, National Cheng Kung University, Tainan, Taiwan; 3 NTU Center of Genomic Medicine, College of Medicine, National Taiwan University, Taipei, Taiwan; 4 Institute of Statistical Science, Academia Sinica, Taipei, Taiwan; 5 Department of Pathology and Graduate Institute of Pathology, College of Medicine, National Taiwan University, Taipei, Taiwan; 6 Division of Chest Medicine, Department of Internal Medicine, Taichung Veterans General Hospital, Taichung, Taiwan; 7 Institute of Biomedical Sciences, Academia Sinica, Taipei, Taiwan; 8 Graduate Institute of Medical Genomics and Proteomics, College of Medicine, National Taiwan University, Taipei, Taiwan; 9 Department of Internal Medicine, College of Medicine, National Taiwan University, Taipei, Taiwan; Vanderbilt University Medical Center, United States of America

## Abstract

The motor protein kinesin superfamily proteins (KIFs) are involved in cancer progression. The depletion of one of the KIFs, KIF14, might delay the metaphase-to-anaphase transition, resulting in a binucleated status, which enhances tumor progression; however, the exact correlation between KIF14 and cancer progression remains ambiguous. In this study, using loss of heterozygosity and array comparative genomic hybridization analyses, we observed a 30% loss in the regions surrounding KIF14 on chromosome 1q in lung adenocarcinomas. In addition, the protein expression levels of KIF14 in 122 lung adenocarcinomas also indicated that approximately 30% of adenocarcinomas showed KIF14 down-regulation compared with the expression in the bronchial epithelial cells of adjacent normal counterparts. In addition, the reduced expression of KIF14 mRNA or proteins was correlated with poor overall survival (P = 0.0158 and <0.0001, respectively), and the protein levels were also inversely correlated with metastasis (P<0.0001). The overexpression of KIF14 in lung adenocarcinoma cells inhibited anchorage-independent growth *in vitro* and xenograft tumor growth *in vivo*. The overexpression and silencing of KIF14 also inhibited or enhanced cancer cell migration, invasion and adhesion to the extracellular matrix proteins laminin and collagen IV. Furthermore, we detected the adhesion molecules cadherin 11 (CDH11) and melanoma cell adhesion molecule (MCAM) as cargo on KIF14. The overexpression and silencing of KIF14 enhanced or reduced the recruitment of CDH11 in the membrane fraction, suggesting that KIF14 might act through recruiting adhesion molecules to the cell membrane and modulating cell adhesive, migratory and invasive properties. Thus, KIF14 might inhibit tumor growth and cancer metastasis in lung adenocarcinomas.

## Introduction

The kinesin (KIF) superfamily consists of motor proteins that transport organelles, proteins and mRNA in an adenosine-5′-triphosphate (ATP)- and microtubule-dependent manner [1], [2]. There are more than 45 members of the KIF superfamily, which are divided into 13 subfamilies according to their orientation, domain and function [3]. KIF14, a member of the kinesin-3 family, contains a motor and a forkhead-associated domain, and this protein plays an important role in cytokinesis and in the segregation, congression and alignment of chromosomes [4]–[6]. Depletion of KIF14 might cause a time delay in the metaphase-to-anaphase transition, inhibit cytokinesis and produce a binucleated status [7], [8]. Further studies have shown that KIF14 might promote efficient cytokinesis through interactions with citron kinase (CIK) and the protein regulator of cytokinesis 1 (PRC1) [7].

Cytokinesis is the final stage of the cell cycle in which the two daughter cells completely separate [9]. Increasing evidence suggests that aberrant cytokinesis might generate unstable tetraploid cells, leading to aneuploidy, which eventually develops into carcinogenesis [9]–[11]. Many components that regulate cytokinesis have been identified, and some molecules, including KIFs, have been clearly associated with cancer. For example, KIF2 and KIF15 have been identified as tumor antigens in breast cancer [12], [13]; KIF13A is overexpressed in some retinoblastomas [14]; KIF20A is up-regulated in pancreatic cancer [15]; KIF3 and KIF4 have been identified as tumor suppresser genes in gastric cancer [16], [17]; and the expression of KIF10 is reduced in hepatocellular carcinoma [18]. In addition, it has been reported that some KIF family members, including KIF3, KIF4 and KIF22, have conflicting roles in tumorigenesis [16], [17], [19]–[23].

Lung cancer is the most common cause of cancer death, especially in Asia, and accounts for 17% of the total cancer deaths worldwide [24], [25]. Recent studies have focused on the role of kinesin proteins, such as KIF3, KIF5B and KIF14, in lung cancer progression; however, the detailed mechanisms underlying the functions of these proteins remain unclear [16], [26]–[28]. Therefore, the aim of this study was to investigate the role of kinesin motor proteins in lung adenocarcinoma.

In this study, we evaluated the potential role of KIF14 in lung adenocarcinoma using loss of heterozygosity (LOH) and array comparative genomic hybridization (CGH) analyses of 138 lung adenocarcinomas in chromosome 1q, which is where KIF14 is located. In addition, we also examined the protein expression of KIF14 in tissue specimens from 122 lung adenocarcinoma patients using immunohistochemical staining. The KIF14 mRNA and protein expression were correlated with the overall survival and metastasis rates in these patients. We also dissected the molecular mechanism of KIF14, which mediates the suppression of cancer cell invasion, migration and adhesion. These results suggest that KIF14 might inhibit tumor growth and cancer metastasis in lung adenocarcinoma.

## Results

### KIF14 Expression in Lung Cancer Patients

**Figure 1:**
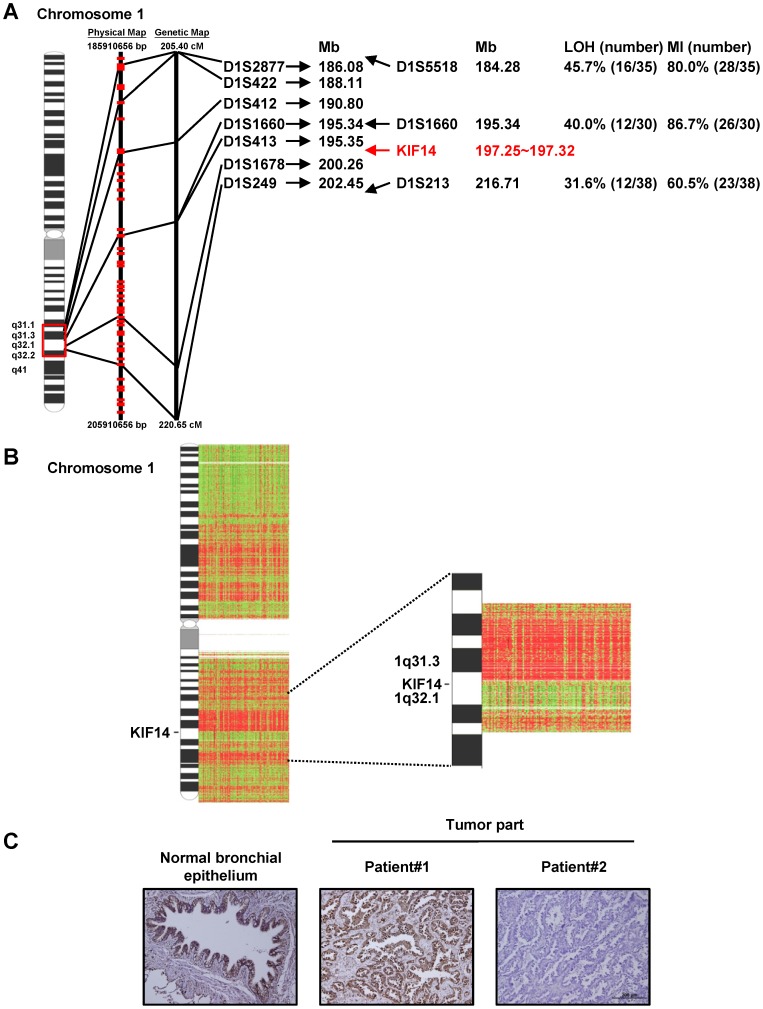
The down-regulation of KIF14 occurred in lung adenocarcinomas. (A) Loss of heterozygosity (LOH) and microsatellite instability (MI) analyses near the location of KIF14 in primary lung adenocarcinoma. Chromosome 1 ideogram showing the microsatellite markers mapped on 1q according to the location database; the patient number and percentage are indicated. (B) The copy-number alteration profiles of chromosome 1. The KIF14 location is indicated within chromosome 1q32.1. (C) The protein expression pattern of KIF14 was examined using immunohistochemistry with anti-KIF14 antibodies in normal bronchial mucosa (left panel) and primary tumor specimens (middle and right panel). Original magnification 200×.

To evaluate the potential role of kinesin in lung adenocarcinoma, we first examined the frequency of LOH in chromosomes using microsatellite markers, and observed that LOH occurred between markers D1S1660 (40.0% LOH and 86.7% microsatellite instability (MI)) and D1S213 (31.6% LOH and 60.5% MI), which are located near KIF14 on chromosome 1q (Figure 1A). To confirm these results, we conducted high-density array CGH in an independent cohort of 138 lung adenocarcinomas [29]. The data indicated that nearly 25% (22.5% and 26.8%) loss from the two probes within KIF14 localization and 5% (2.9% and 6.5%) gain occurred in the patients (Figure 1B and Table 1).

**Table 1:** The array CGH and KIF14 protein expression in lung adenocarcinomas and adjacent normal bronchial epithelia.

Methods	Parameter	No. patients	Percentage
Array CGH^1^	Normal	97/98	70.3/71.0
	Loss	37/31	26.8/22.5
	Gain	4/9	2.9/6.5
	Total	138	100.0
Immunohistochemistry^2^	0	63	51.6
	1	22	18.0
	2	37	30.3
	Total	122	100.0

1 There are two probes (chr1∶198737634–198793986/chr1∶198800168-198856322 [HG18]) located in KIF14.

2 0 denotes that the immunoreactivity of KIF14 antibodies was almost equal between normal bronchial mucosa and tumors, 1 denotes that the immunoreactivity was up-regulated in tumors compared with normal bronchial mucosa and 2 denotes that the immunoreactivity was down-regulated in tumors compared with normal bronchial mucosa.

Abbreviations: CGH, comparative genomic hybridization.

We next examined the expression of KIF14 in tissue specimens and adjacent normal tissues from 122 lung adenocarcinomas using immunohistochemistry. The specificity of the KIF14 antibody was confirmed through KIF14 antigen competition using immunoblotting and immunohistochemistry (Figure S1), and the clinical characteristics of these patients are summarized in Table 2. These results showed that KIF14 was strongly expressed in normal bronchial epithelial cells (Figure 1C). Further analysis indicated that 30.3% of the adenocarcinomas showed KIF14 down-regulation in the tumors compared with the bronchial epithelial cells in adjacent normal counterparts and that 18.0% of the adenocarcinomas showed a higher KIF14 expression than in the normal bronchial mucosa (Table 1). Taken together, these results suggest that KIF14 might have a greater loss (∼30%) than gain (3–6%) in lung adenocarcinomas.

**Table 2:** Characteristics of the 122 lung adenocarcinomas determined using immunohistochemistry staining^1^.

Parameter	Number	Low KIF14(%)	High KIF14(%)	P
Number of patients	122	63 (51.6)	59 (48.4)	
Age (means ± SD)	64±10.7	64.9±9.9	62.1±11.4	0.153^2^
Sex				
Male	53	30 (47.6)	23 (39.0)	
Female	69	33 (52.4)	36 (61.0)	0.336
Tumor size				
≤3 cm	56	26 (41.3)	30 (50.8)	
>3 cm	66	37 (58.7)	29 (49.2)	0.289
Lymph node metastasis				
Negative	88	38 (60.3)	50 (84.7)	
Positive	34	25 (39.7)	9 (15.3)	0.003
Differentiation				
Well	30	10 (15.9)	20 (33.9)	
Moderate	91	52 (82.5)	39 (66.1)	
Poor	1	1 (1.6)	0 (0.0)	0.048
Tumor stage				
Stage I	86	37 (58.7)	49 (83.0)	
Stage II	16	8 (12.7)	8 (13.6)	
Stage III–IV	20	18 (28.6)	2 (3.4)	0.001

1 KIF14 expression was designated as ‘high’ or ‘low’ using “70% immunoreactivity in tumor sections” as the cut-off point. The P values were calculated using a two-sided Pearson’s chi-squared test.

2 The P values were calculated using Student’s *t*-test.

### Low Expression of KIF14 was Associated with Poor Overall Survival in Lung Adenocarcinoma Patients

**Figure 2:**
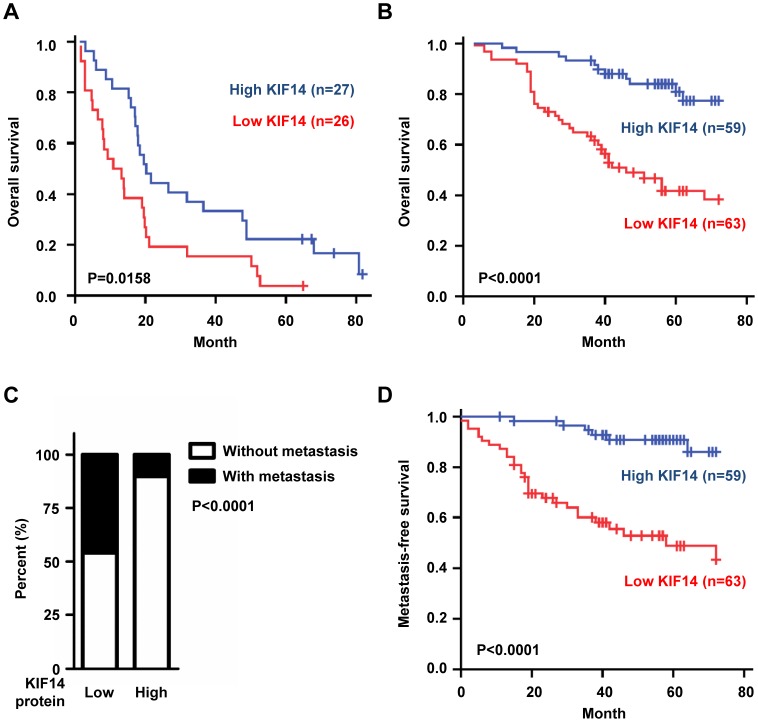
The expression of KIF14 was negatively correlated with clinical outcomes in the lung adenocarcinoma patients. (A) Low KIF14 mRNA expression was associated with poor overall survival in the patients with lung adenocarcinoma. The KIF14 mRNA levels were measured using real-time quantitative RT-PCR of primary tumor specimens. The median value was used to divide patients into low (red line) and high (blue line) KIF14 expression groups (log rank, P = 0.0158). (B) Low KIF14 protein expression was associated with poor overall survival in an independent cohort of lung adenocarcinoma patients. The patients were divided into two groups according to the immunoreactivity of the KIF14 antibodies. The cut-off value was 70% in tumor sections showing immunoreactivity to the antibodies. Kaplan-Meier analyses were used to estimate the overall survival in the lung adenocarcinoma patients according to KIF14 expression (log rank, P<0.0001). (C) Percentage analysis of the metastatic status in high and low KIF14 expression groups. Patients in both high and low KIF14 expression groups were further divided based on the presence of metastasis; and the numbers of each subgroup are shown as a percentage using statistical analysis (chi-square test, P<0.0001). (D) The KIF14 expression in human lung adenocarcinoma predicted the occurrence of metastasis. Metastasis-free survival curves of 122 lung adenocarcinoma patients stratified by low (red line) and high (blue line) KIF14 protein expression (log rank, P<0.0001).

To further investigate whether the expression of KIF14 in lung adenocarcinoma is correlated with clinical outcomes, the mRNA levels of KIF14 in tumor specimens from 53 lung adenocarcinoma patients were determined using the real-time quantitative reverse transcriptase polymerase chain reaction. The clinical characteristics of these patients are summarized in Table S1. Patients with low KIF14 expression exhibited worse overall survival than those with high KIF14 expression (Figure 2A, P = 0.0158). Multivariate Cox proportional hazard regression analyses indicated that KIF14 expression (hazard ratio [HR] = 0.341, 95% confidence interval [CI] = 0.179–0.652; P = 0.0011) and disease stage (HR = 1.962, 95% CI = 1.311–2.936; P = 0.0010) were independent factors associated with the overall survival of lung adenocarcinoma patients (Table S2).

To extend our analysis to protein expression levels, we used immunohistochemistry to examine the expression of KIF14 in tumor specimens from 122 lung adenocarcinoma patients in an independent cohort. Consistent with our previous results, patients with low levels of KIF14 expression had poorer overall survival than patients with high levels of KIF14 expression (P<0.0001; Figure 2B). The multivariate Cox proportional hazard regression analyses revealed that the independent prognostic factors were KIF14 expression (HR = 0.37, 95% CI = 0.18–0.76; P = 0.0006) and disease stage (HR = 5.38, 95% CI = 2.82 to 9.88; P<0.0001) (Table 3).

**Table 3:** Hazard ratios for death (from any cause) among patients with lung adenocarcinoma determined using immunohistochemistry staining according to multivariate Cox regression analysis^1^.

Parameter	Hazard ratio (95% CI)	P
KIF14 expression	0.37 (0.18 to 0.76)	0.006
Sex	0.99 (0.55 to 1.78)	0.973
Age	1.02 (0.99 to 1.05)	0.239
Stage	5.38 (2.82 to 9.88)	<0.0001

1 KIF14 expression was designated as ‘high’ or ‘low’ using “70% immunoreactivity in tumor sections” as the cut-off point (low KIF14 as the referent), and these values were adjusted according to sex (female as the referent vs. male), age and tumor stage (according to TNM classification). The P values (two-sided) were calculated using Pearson’s chi-square test.

Abbreviations: CI, confidence interval.

### KIF14 was Negatively Correlated with Metastasis in Lung Adenocarcinoma Patients

We further investigated whether the protein expression levels of KIF14 in tumor specimens were negatively correlated with cancer metastasis in patients. The results from 122 lung adenocarcinomas showed that 29 of the 63 patients with low KIF14 protein expression had cancer metastasis. However, only 6 of the 59 patients with high KIF14 expression developed cancer metastasis (Figure 2C, P<0.0001). The Kaplan-Meier estimate of metastasis-free-survival also showed that patients with low KIF14 expression had a significantly poorer metastasis-free survival rate than those with high KIF14 expression (Figure 2D, P<0.0001). These results indicate that KIF14 might be a tumorigenic and metastatic suppressor in lung adenocarcinoma.

### Overexpression of KIF14 in Lung Adenocarcinoma Cells Inhibited Anchorage-independent Growth in vitro and Xenograft Tumor Formation *in vivo*

**Figure 3:**
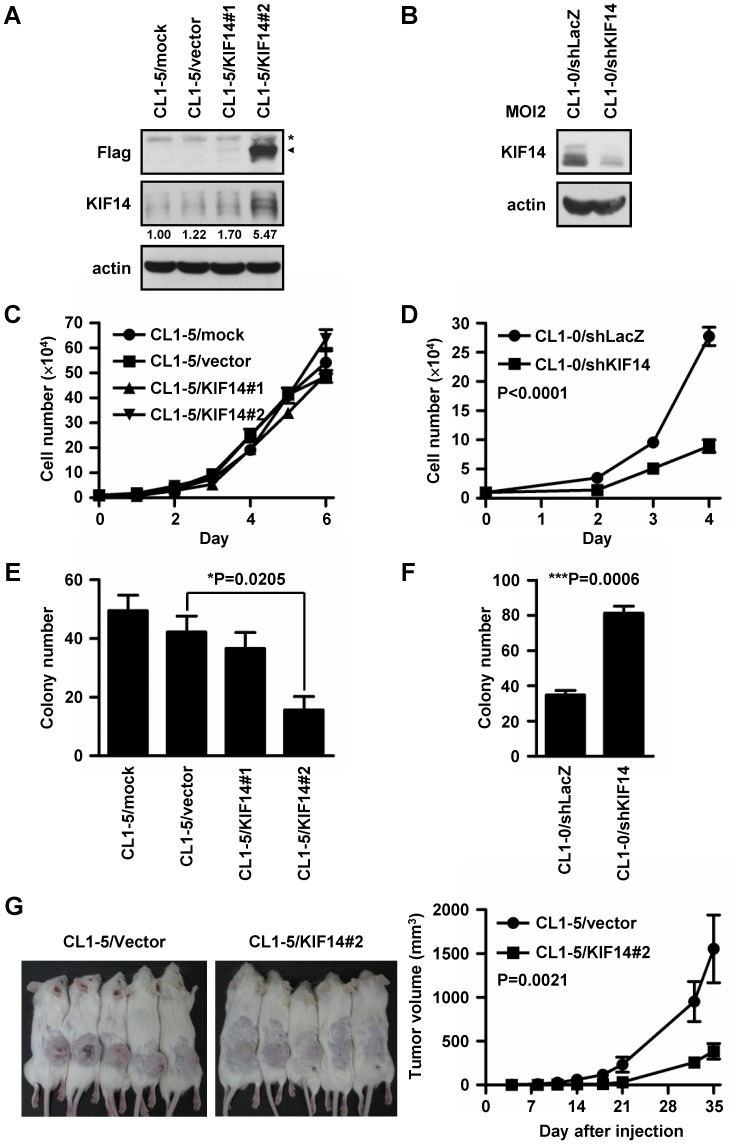
KIF14 inhibited anchorage-independent growth *in vitro* and tumor growth *in vivo*. (A) Cell lines constitutively expressing KIF14 were established through the transfection of pCMV-7.1-3-3×Flag-KIF14 into CL1-5 cells. Single colonies were selected using Geneticin, and the KIF14 protein expression was assessed through immunoblotting with anti-Flag antibodies; actin was used as an internal control. The asterisk and arrowhead indicate the non-specific band and Flag-KIF14, respectively. (B) KIF14 expression was knocked down in CL1-0 cells using shRNA lentiviral infection. After selection with puromycin for two weeks, the KIF14 protein expression patterns were assessed through immunoblotting with anti-KIF14 antibodies; actin was used as an internal control. (C and D) Effects of KIF14 overexpression and silencing on cultured tumor cell proliferation. The cell number was calculated at the indicated times after planting. The error bars represent standard deviations of the mean. No significant differences were observed in the proliferation rates between different cell lines by one-way ANOVA analysis (C). (E and F) Anchorage-independent growth of KIF14-overexpressing and -silencing cell lines were assessed by colony formation in soft agar. The error bars represent the standard deviations of three independent experiments performed in triplicate. (G) KIF14 overexpression reduced tumor growth. The mice were injected subcutaneously into the flanks with CL1-5/vector and CL1-5/KIF14#2 cells. Left panel: Final tumor photographs at 35 days post-subcutaneous injection. Right panel: Average tumor volume at 35 days post-subcutaneous injection was calculated, and the animals were subsequently sacrificed. The bars represent the means ± standard deviations of six mice per group (one-way ANOVA, P = 0.0021).

Our observations supported the hypothesis that KIF14 acts as a tumor suppressor in lung adenocarcinoma. To examine this hypothesis further, we first assessed the endogenous KIF14 protein expression in low-invasive CL1-0 and high-invasive CL1-5 lung adenocarcinoma cells (Figure S2A). We observed that KIF14 protein in CL1-0 cells was higher than that in CL1-5 cells. To examine the changes in cancer progression *in vitro* and *in vivo* [30], we established stable Flag-tagged KIF14-expressing cell lines from CL1-5 cells and KIF14-silenced CL1-0 cells, and the protein expression pattern was confirmed through immunoblotting (Figure 3A and 3B). In the detection of cell proliferation rate, we observed no significant differences between vector-only controls and the KIF14-overexpressing cell lines (Figure 3C), but reduced proliferation was observed in the KIF14-silenced cells compared with the shLacZ control (Figure 3D, P<0.0001). Based on evidence showing that anchorage-independent cell growth might be associated with tumorigenic potential [31], [32], we further examined the growth of the stable cell lines in soft agar. The results indicated that the number of colonies formed was reduced when KIF14 was overexpressed in CL1-5 cells (Figure 3E) and increased when KIF14 was silenced in CL1-0 cells (Figure 3F). Subsequently, the stable cell lines were subcutaneously transplanted into SCID mice to explore the effect of KIF14 on tumor growth *in vivo*. The overexpression of KIF14 significantly reduced tumor growth compared with the controls (Figure 3G, right; P = 0.0021). The representative pictures of CL1-5/vector- and KIF14#2-inoculated mice are shown (Figure 3G, left).

### Manipulation of KIF14 Expression Levels Altered the Migration, Invasion and Adhesion of Lung Adenocarcinoma Cell Lines

**Figure 4:**
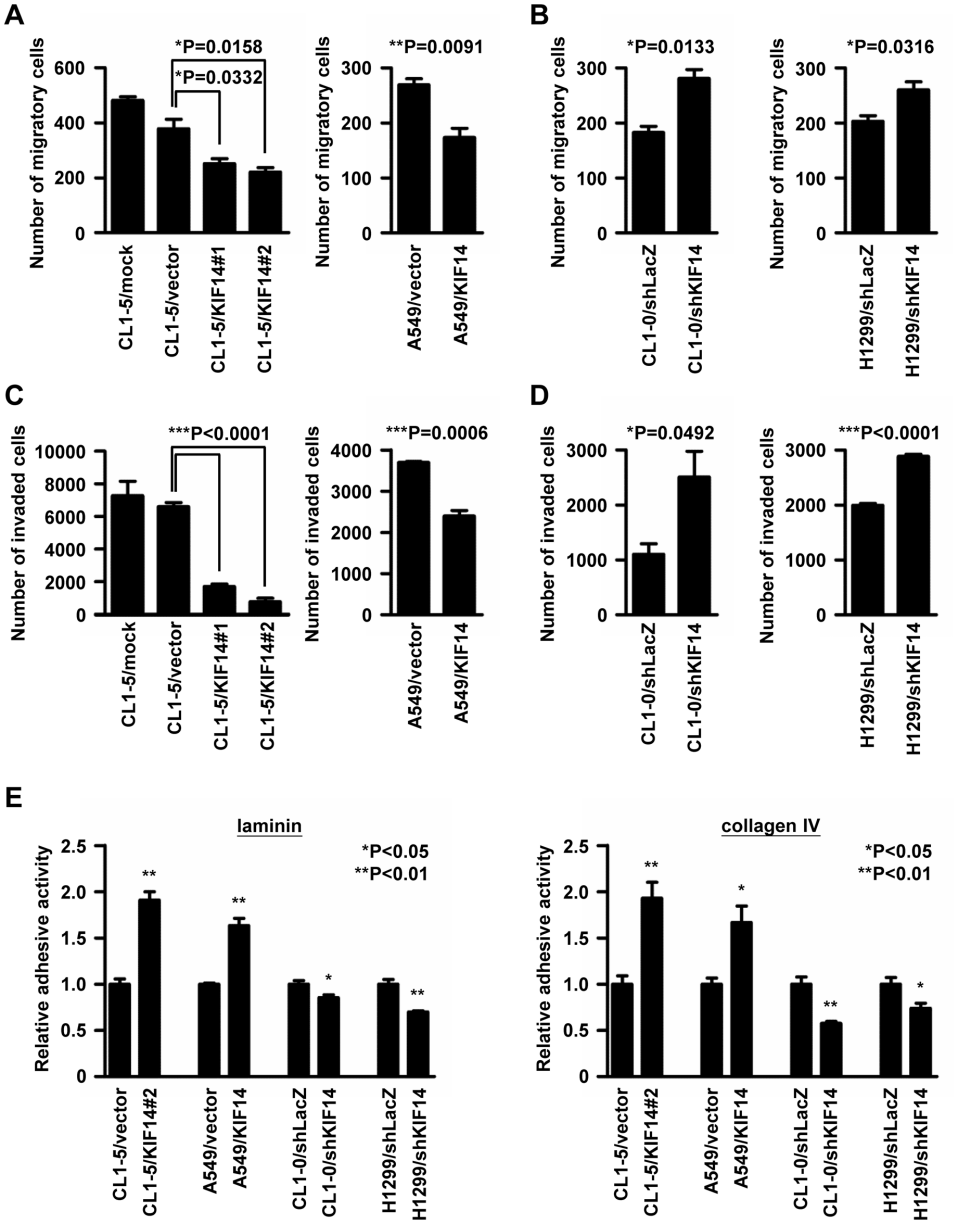
Manipulating the expression of KIF14 inhibited cell migration, invasion and adhesion. (A) CL1-5 cells with permanent KIF14 overexpression and A549 cells transiently infected with lentivirus encoding non-tagged full-length KIF14 were analyzed for the cell migratory ability in a wound-healing assay. The data show the number of migratory cells, and the P values were calculated relative to vector-only controls using Student’s *t*-test. (B) Wound-healing assay of CL1-0 and H1299 cells with KIF14 silencing. The number of migratory cells was also calculated. (C and D) KIF14 overexpression reduced cell invasion. The invasion ability of stable or transiently infected KIF14 and KIF14-silenced cell lines was measured using a modified Boyden chambers assay. The invading cells were indicated with propidium iodide staining and quantified (n = 3). (E) KIF14 overexpression enhanced cell adhesion. A 96-well plate was coated with laminin and collagen IV. A total of 3×10^4^ cells were plated onto a 96-well plate, incubated at 37°C for 1 hour and the adhesive activity was calculated using crystal violate staining. High absorbance at 540 nm indicated enhanced cell adhesion.

Because the expression of KIF14 was negatively correlated with metastasis in patients, we next investigated whether KIF14 affected cancer cell metastasis. A wound-healing assay was used to detect the migration abilities of KIF14-expressing cells. KIF14 expression reduced the migration of CL1-5 cells, whereas the knockdown of KIF14 protein expression in CL1-0 cells increased cell migration (all P<0.05; Figure 4A and 4B, left). To confirm the results obtained from the CL cells, we examined the KIF14 expression levels in other lung adenocarcinoma cell lines (Figure S2B) and overexpressed KIF14 in A549 cells with low endogenous KIF14 protein and silenced KIF14 in H1299 cells with relative high KIF14 protein levels. The KIF14 protein expression and proliferation rates of these cells are shown in Figure S3. Similar results were obtained using additional cell lines compared with CL cells (all P<0.05; Figure 4A and 4B, right).

We next assessed the invasive properties of these cell lines using modified Boyden chamber assays. KIF14 overexpression in CL1-5 and A549 cells reduced cell invasiveness 11.6 to 64.9% compared with the vector-only controls (P<0.05; Figure 4C). Conversely, the knockdown of endogenous KIF14 expression in CL1-0 and H1299 cells increased cell invasiveness 1.4- to 2.3-fold (P<0.05; Figure 4D).

In general, cell adhesion is a complex mechanism involved in a variety of processes, including cell migration and invasion [33]. Because these data indicated that the expression of KIF14 could affect the migratory and invasive abilities of lung adenocarcinoma cell lines, we further investigated whether cell adhesion was affected after modulating KIF14. Consistent with our hypothesis, the data showed that the overexpression of KIF14 increased adhesion to the extracellular matrix proteins, laminin and collagen IV, in both CL1-5 and A549 cells; moreover, silencing KIF14 reduced adhesion in both CL1-0 and H1299 cells (Figure 4E).

### KIF14 Might Regulate the Recruitment of Adhesive Molecule CDH11 to the Cell Membrane in Lung Adenocarcinoma Cell Lines

**Figure 5:**
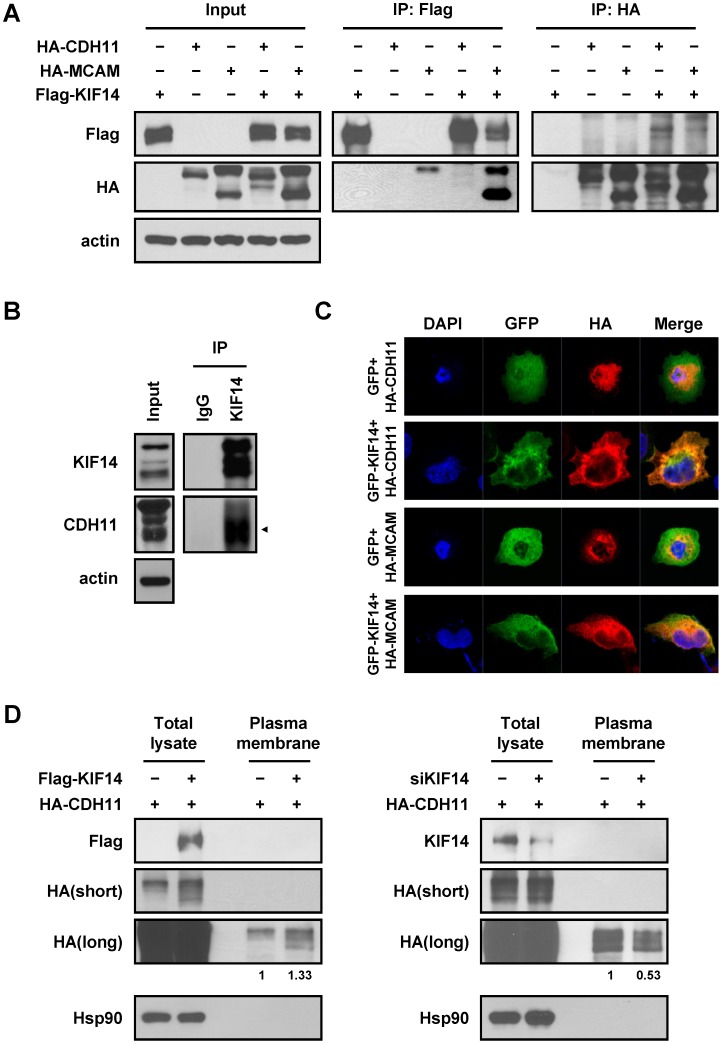
KIF14 modulated the distribution of the cargo molecules. (A) KIF14 associated with CDH11 and MCAM. The lysate of HEK293T cells transfected with the indicated plasmids was used in exogenous immunoprecipitation assays. Immunoblotting was performed with the indicated antibodies. Actin was used as an internal control. (B) The lysate of H460 cells was used in endogenous immunoprecipitation assays. Immunoblotting was performed with the indicated antibodies. (C) The distribution of CDH11 and MCAM changed with the overexpression of KIF14. H1299 cells were co-transfected with HA-CDH11 or HA-MCAM and GFP-KIF14, fixed and hybridized using anti-HA antibodies. The signal was captured using a confocal microscope (original magnification, 1,000×). (D) HEK293T cells were co-transfected with HA-CDH11 and Flag-KIF14 or siKIF14 and subsequently, the membrane fraction was isolated. The protein in the membrane fraction and total cell lysate was analyzed through immunoblotting. The amounts of HA-CDH11 in the membrane fraction were quantified through normalization with the amount in the total cell lysates. Hsp90 was used as a cytosol marker.

Because KIF14 inhibited cell migration, invasion and adhesion *in vitro* and cancer metastasis *in vivo*, we wanted to determine whether changes in the motor function in cells could lead to physiological changes. Hence, we investigated potentially associated partners of KIF14 using the String 9.0 protein interaction database (http://string.embl.de/) and identified the two known associated proteins, PRC1 and CIK [7], and two additional proteins, cadherin 11 (CDH11) and melanoma cell adhesion molecule (MCAM), which were potentially associated with KIF14 (Figure S4). Because CDH11 and MCAM are important adhesion molecules associated with tumorigenesis and metastasis [34], [35], we hypothesized that KIF14 might affect cell adhesion and migration through these cargo molecules. To confirm this hypothesis, we first examined whether KIF14 could associate with CDH11 and MCAM using co-immunoprecipitation. The results showed that HA-tagged CDH11 and MCAM associated with Flag-tagged KIF14 proteins (Figure 5A). Endogenous immunoprecipitation also confirmed associations between KIF14 and CDH11 *in vivo* (Figure 5B). The distributions of KIF14 and CDH11 or MCAM proteins were examined in H1299 cells using immunofluorescence staining. The results showed that CDH11 and MCAM proteins might co-localize in common compartments with KIF14 protein, and the expression of CDH11 and MCAM was primarily observed at the cell periphery when KIF14 was overexpressed (Figure 5C). As KIF14 is a motor protein that participates in the transport of molecules, we further explored whether the expression of KIF14 could regulate the localization of CDH11. We isolated membrane fraction proteins and analyzed the expression of CDH11 from KIF14-overexpressing and KIF14-silenced cell lines. The amounts of HA-CDH11 in the membrane fraction were quantified through normalization with the amount in total cell lysates, and the results showed that the overexpression of KIF14 increased the expression of CDH11 in the membrane fraction compared with the control cells (Figure 5B, left). In contrast, the depletion of KIF14 reduced CDH11 expression at the cell surface compared with the non-silenced controls (Figure 5B, right). We also examined the distribution of endogenous CDH11 in CL1-5/vector, CL1-5/KIF14#2, CL1-0/shLacZ and CL1-0/shKIF14 cells, and the results were similar to previous data (Figure S5).

## Discussion

These results demonstrated that the motor protein KIF14 acts as a tumor and metastasis suppressor in lung adenocarcinoma. We observed more loss than gain near KIF14 on chromosome 1q32 (Figure 1) and found that patients with low KIF14 expression exhibited worse overall and metastasis-free survival compared with patients expressing high levels of KIF14 (Figure 2). The overexpression of KIF14 in lung cancer cells significantly inhibited anchorage-independent growth *in vitro* and xenograft tumor formation *in vivo* (Figure 3). Furthermore, modulating the expression of KIF14 inhibited cell migration, invasion and adhesion through associating with and increasing the membrane expression of the cargo protein, CDH11 (Figure 4 and 5). Therefore, KIF14 might be a potential target for lung adenocarcinoma treatment.

Chromosome mis-segregation results in aberrant cytokinesis and aneuploidy. This phenomenon is prevented in normal cells through cell cycle arrest and apoptosis; however, tumor cells show increasing rates of aneuploidy, which might be associated with poor clinical outcomes [36]–[41]. KIF14 functions in chromosome segregation and cytokinesis, and its depletion might result in a binuclear or multi-nuclear status [4], [5], [7], [8]. These results support the hypothesis that aneuploidy develops into carcinogenesis [9]–[11], [42]. In this study, the overexpression or silencing of KIF14 in cells reduced or increased, respectively, anchorage-independent growth *in vitro*, and KIF14 overexpression reduced tumor growth *in vivo* (Figure 3). These results are consistent with those of a previous study in which the silenced expression of KIF14 in pancreatic cancer cells resulted in increased anchorage-independent survival [43]. Apart from KIF14, many other cytokinesis regulators have also been reported as tumor suppressor genes, including KIF1A, KIF1B, KIF3, KIF4, KIF10, Von Hippel-Lindau syndrome protein (pVHL) and BRCA2 [16]–[18], [44]–[47]. Taken together, these results suggest that a KIF14 deficiency might promote aneuploidy, resulting in tumor formation, and that KIF14 might act as a tumor suppressor in lung cancer.

Cancer progression is a complex process involving cell growth, basement membrane degradation, cell migration, invasion, adhesion, and metastasis [48], [49]. The results obtained in the present study showed that KIF14 was not only involved in tumorigenesis but also participated in cell adhesion, migration and invasion (Figure 3 and 4). Immunochemistry indicated that the expression levels of KIF14 were negatively correlated with cancer metastasis in patients (Figure 2C and 2D). In CL cell lines, the KIF14 protein levels were inversely associated with cell invasive abilities (Figure S2A). These observations were similar to those of a previous report in which KIF14 expression was negatively correlated with Matrigel and nerve invasion in pancreatic cancer [43]. Therefore, KIF14 might act as a metastatic suppressor in lung adenocarcinoma.

Notably, the results of the *in vitro* assays were slightly discordant. The data obtained in the present study suggest that KIF14 silencing reduces cell proliferation (Figure 3), potentially reflecting M phase delays and cytokinesis failures. Nevertheless, KIF14 overexpression did not significantly affect cell proliferation compared with the control. We further examined the distribution of KIF14 and the phenomena of cells in each phase, and the data indicated that no specific defect occurred with KIF14 overexpression (unpublished data). Thus, the silencing or overexpression of KIF14 reduced or did not affect cell proliferation but increased or reduced colony formation; such effects are regarded as cell transforming abilities. The overexpression of KIF14 (KIF14#2) in CL1-5 cells strongly reduced colony formation, cell migration and invasion, and the slight expression of KIF14 (KIF14#1) mildly reduced colony formation and strongly reduced cell migration and invasion (Figure 3 and 4). These results suggested that KIF14 protein affects cell transformation and invasion to different degrees. We propose that the slight increase of KIF14 protein expression in CL1-5/KIF14#1 stable cells also promotes dramatic cell migration by inhibiting the transport of several important cargo proteins involved in cell migration and invasion.

Although KIF14 is an important motor protein that participates in cytokinesis through interactions with PRC1 and citron kinase, the role of KIF14 in other functions remains unclear [7]. The results obtained in the present study showed that KIF14 might regulate cell migration and adhesion through associations with CDH11 and MCAM; this increase in the membrane localization of CDH11 in lung adenocarcinoma cell lines is a novel finding with regard to the function of KIF14 (Figure 5). These results are similar to those of a report in which KIF13 participated in mannose-6-phosphate receptor transport from the cytosol to plasma membrane [50]. These data indicate that KIFs might regulate different cellular functions by changing the localization of different cargo proteins.

These results support the hypothesis that KIF14 acts as a tumor suppressor and metastasis inhibitor in lung adenocarcinoma. The role of KIF14 in cancer progression has been discussed in recent years. Corson et al., Kim et al. and Wang et al. showed that KIF14 is highly expressed in retinoblastoma, breast cancer, ovarian cancer, hepatocellular carcinoma, glioma and some lung tumors [28], [51]–[55]. Using CGH array analyses, Corson et al. observed a 20% gain in lung and bronchus tumors in a large area of chromosome 1q31-1q32 [51]; however, in the present study, we conducted CGH array analyses and observed a 25% (22.5% and 26.8%) loss from the two probes within KIF14 localization and a 5% (2.9% and 6.5%) gain in lung adenocarcinomas. To explore this discrepancy, we also examined the mRNA expression of KIF14 in our study cohort using the primers and probe from Corson et al.’s studies. Similar to the results shown in Figure 1B, patients with low KIF14 expression exhibited worse overall survival compared to patients with high KIF14 expression. Corson et al. also silenced KIF14 using siRNA in lung tumor cell lines and indicated that KIF14 silencing reduced cell proliferation and colony formation [28]. In the present study, we silenced the expression of KIF14 through the lentiviral delivery of shKIF14 and obtained similar results for proliferation but opposite results with regard to colony formation. Interestingly, it remains unclear why different results were obtained for the same gene; however, there are several possible explanations for these phenomena. 1. Difference in KIF14 protein tags: Theriault et al. utilized C-terminal EGFP- and Myc-tagged KIF14 in proliferation and colony formation assays, and EGFP-tagged KIF14 exhibited milder effects than Myc-tagged KIF14 [54]. Therefore, we generated non-tagged wild-type KIF14 constructs and examined the effects on cell invasiveness; the results were similar to those of Flag-tagged KIF14 (Figure 4C). We also generated C-terminal Flag-tagged KIF14 to examine cell migration, and the data were similar to those obtained with N-terminal Flag-tagged and non-tagged KIF14 (data not show). 2. Ethnic differences: a recent study explicitly indicated that the occurrence, cause, gene mutation status, survival, prognosis, and lung cancer treatment were different in Asia and the US [56]. 3. One gene or one protein might play dual or multiple roles in the physiological function. For example, transforming growth factor beta (TGF-beta) acts as either a tumor-suppressor or a tumor-promoter under different conditions during cancer progression [57], [58], and signal transducer and activator of transcription 3 (STAT3) has an oncogenic or a tumor suppressor role depending on the mutational background of the tumor [59]. Whether KIF14 has a dual effect remains unknown.

In conclusion, the down-regulation of KIF14 occurred in 30% of lung adenocarcinomas, and the expression of KIF14 was negatively correlated with clinical outcomes in the lung adenocarcinoma patients examined in this study. Thus, KIF14 might inhibit tumor growth and cancer metastasis through controlling the recruitment of adhesion molecules to the cell membrane to modulate cell adhesion, migration and invasion.

## Materials and Methods

### Ethics Statement

The Institutional Review Board of the National Taiwan University Hospital and Taichung Veterans General Hospital approved this investigation, and informed written consent was obtained from all patients involved in this study.

### Patient Specimens

The tissue specimens for the LOH and immunohistochemistry analyses were collected from patients who had undergone surgical resections and been diagnosed with lung adenocarcinoma at the National Taiwan University Hospital (Taipei, Taiwan) from 2003 to 2006. For the real-time quantitative polymerase chain reaction, the tissue sections were obtained from 53 patients who had undergone surgical resection and been diagnosed with lung adenocarcinoma at Taichung Veterans General Hospital (Taichung, Taiwan) from 2000 to 2004. All studies were approved by the Institutional Review Board of the National Taiwan University Hospital and Taichung Veterans General Hospital. None of the patients received pre-operative adjuvant chemotherapy or radiation therapy. The post-surgical pathological stage of each tumor tissue was classified according to the international TNM (tumor-node-metastasis) classification [60].

### Loss of Heterozygous (LOH) and Array Comparative Genomic Hybridization (CGH)

The genomic DNA isolation, LOH analysis using microsatellite markers and array CGH analysis were performed as previously described [29], [61].

### Immunohistochemistry

The paraffin-embedded sections used for the immunohistochemistry of the tumor tissues were collected from patients with lung adenocarcinoma. Deparaffinized 4-µm-thick sections were autoclaved in Trilogy Solution (Cell Marque Corp., Rocklin, California) at 121°C for 10 minutes, treated with 3% H_2_O_2_-methanol, subsequently incubated with DakoCytomation Dual Endogenous Enzyme Block (DakoCytomation Inc., Carpinteria, California) for 10 minutes, Ultra V Block (LAB VISION Corporation, Fremont, California) for 10 minutes, Antibody Dilution Buffer (Ventana Medical Systems Inc., Tucson, Arizona) for 10 minutes, and an anti-KIF14 antibody (diluted 1∶100; Bethyl Laboratories Inc., Montgomery, Texas) overnight at 4°C. The immunoreactive staining was detected using a BioGenex Super sensitive Link-Label Kit IHC detection system (BioGenex, San Ramon, California), according to the manufacturer’s instructions.

### Real-time Quantitative Polymerase Chain Reaction

KIF14 transcript levels were determined through quantitative RT-PCR using an ABI prism 7900 sequence detection system (Applied Biosystems, Foster City, California), according to the manufacturer’s instructions. The predesigned primers and probe set for KIF14 (Hs00208408_m1) were purchased from Applied Biosystem. The TATA-binding protein (TBP) was used as an internal control. The relative amount of tissue KIF14 mRNA was standardized against the geometric mean of TBP mRNA. The median value was used to divide patients into high- and low-expression groups. The experiments were performed in duplicate; no-template controls and standards, which were diluted from KIF14 stable cell lines, were included in each assay. The P value was determined using a 2-sided log-rank test.

### Cell Line and Culture Conditions

A549 (a human adenocarcinoma alveolar basal epithelial cell line), H1299 (a human non-small cell lung carcinoma cell line derived from the lymph nodes), and HEK293T (human embryonic kidney cell line) cells were purchased from the American Type Culture Collection (ATCC, Manassas, Virginia). CL1-5 (Human lung adenocarcinoma cell line) is a cell subline with higher invasiveness, which was selected from CL1-0 using a Transwell invasion assay [62]. EKVS, H23, H460, H522 and Hop62 lung cancer cell lines were purchased from the Developmental Therapeutics Program of the National Cancer Institute (NCI, Bethesda, Maryland). The H441 cell line was a kind gift from Dr. Win-Ping Deng (Institute of Biomedical Materials and Engineering, Taipei Medical University), and PC9 cells were obtained from Dr. Chih-Hsin Yang (Graduate Institute of Oncology, National Taiwan University College of Medicine). The cells were cultured in DMEM or RPMI 1640 supplemented with 10% fetal bovine serum (all from Invitrogen, Eugene, Oregon) and grown in a humidified atmosphere with 5% CO_2_ at 37°C. For the transfection, the cells were seeded onto a tissue culture plate at the density required to achieve 70% confluence. Plasmid DNA was transfected using Lipofectamine 2000 (Invitrogen, Eugene, Oregon), according to the manufacturer’s instructions. The total DNA concentration was adjusted using empty vector plasmid in each transfection experiment.

### Plasmid Constructs

The KIF14 (GenBank™ accession number NM_014875.2) expression plasmids p3xFlag-KIF14 and pLKO-AS2-KIF14 were produced through the ligation of PCR-generated inserts into p3xFlag-CMV-7.1-2 and pLKO-AS2-neo, respectively. Human full-length CDH11 and MCAM (NM_001797.2 and NM_006500.2) were amplified from cDNA and subcloned into the pcDNA3.1-HA3 vector. The shKIF14 plasmids were purchased from the National RNAi Core Facility located at the Institute of Molecular Biology/Genomic Research Center, Academia Sinica (Taipei, Taiwan).

### Stable Cell Lines

The purified plasmid p3xFlag-KIF14 was transfected into 70% confluent CL1-5 cells using Lipofectamine 2000 reagents in a total volume of 1 ml of Opti-MEM (Invitrogen), as previously described [63]. Other CL1-5 cells were transfected with p3xFlag vector containing no insert and were used as controls. Geneticin (Merck, Darmstadt, Germany) was added at a concentration of 450 µg/ml to select for a pooled population of stable transfectants, and the selection medium was changed every 3 days for a 3-week period. Clones of resistant cells derived from a single cell were isolated and proliferated for further characterization.

### Lentivirus Production and Infection

HEK293T cells were transfected with pLKO.1-ShLacZ or pLKO.1-shKIF14 together with two helper plasmids, pCMVΔR8.91 and pMD.G, using Lipofectamine 2000 Transfection Reagent, according to the manufacturer’s instructions. The viruses were harvested at 24, 48, and 72 hours after transfection. The virus was filtered using a 0.45 µm low-protein-binding filter and frozen at −80°C. The cells were transduced in the presence of polybrene (Sigma) with lentiviral particles at a multiplicity of infection (MOI) of 2.

### Cell Proliferation

The cell number was analyzed using the trypan blue dye exclusion method. Briefly, 1×10^4^ cells per well were plated in 12-well plates and counted at the indicated time periods. Subsequently, the cells were trypsinized. An aliquot of 20 µl of cell suspension was added to an equal volume of 0.4% trypan blue. The cell mixture was transferred to the edge of a hemocytometer, and the viable and non-viable cells were counted under a microscope.

### Colony Formation

The cells were seeded at 1×10^3^ cells/well in 6-well culture dishes in suspensions of 0.35% agar containing medium supplemented with 10% fetal bovine serum on top of a bed of 0.7% agar containing the same medium. The plates were placed in the incubator for 4 weeks, washed with PBS and fixed with 4% paraformaldehyde at room temperature for 10 minutes. After washing again with PBS, the cells were stained with 0.1% crystal violet, and the colonies were counted using the naked eye. The average numbers of colonies for both the control and experimental groups were calculated. All experiments were performed in triplicate.

### Xenograft Tumor Growth *in* *vivo*

For the *in vivo* xenograft tumor growth assay, a single-cell suspension containing 10^6^ cells (including CL1-5/vector and CL1-5/KIF14-overexpressing cells) in 0.1 ml of PBS was injected into the subcutaneous space of 6-week-old severe combined immunodeficiency (SCID) mice (BioLASCO Taiwan Co., Ltd, Taipei, Taiwan). The tumor sizes were detected every 3 days post injection (n = 6 per group), and the total volume was calculated using the formula a*b^2^/2, where a is the largest diameter (mm) and b is the smallest diameter (mm). All mouse experiments were performed in accordance with the animal guidelines of the Department of Animal Care at the Institute of Biomedical Sciences (Academia Sinica, Taipei, Taiwan).

### Scratch Wound-healing Assay

The cells were seeded onto 6-well tissue culture dishes and grown to confluence. Each confluent monolayer was wounded linearly using a pipette tip and washed 3 times with PBS. Thereafter, the cell morphology and migration were observed and photographed at regular intervals for 12 hours. Three independent experiments were performed in triplicate for each cell line.

### Modified Boyden Chamber Invasion Assay

The materials and protocol for the chamber invasion assay were described in a previous paper [63].

### Adhesion Assay

The 96-well plates were coated with 5 µg/ml laminin or 80 µg/ml collagen IV and incubated overnight at 4°C, washed 3 times with PBS, blocked with 1% BSA in PBS at 37°C for 1 hour and washed 3 times with PBS again. The cells (3×10^4^) were added into each well at 37°C for 40 minutes. The unbound cells were removed by washing with PBS, and the bound cells were stained with 0.1% crystal violet. The dye was resolved in 100 µl methanol, and the absorbance was measured at 540 nm.

### Isolation of Plasma Membrane Protein

Cell membrane proteins were isolated from whole cells using the Plasma Membrane Protein Extraction Kit (Biovision, Mountain View, California), according to the manufacturer’s instructions.

### Immunoprecipitation and Immunoblotting Assay

Immunoprecipitation and Immunoblotting were performed as previously described [63]. The cells were transfected with the indicated plasmids. At 36 hours after transfection, the cells were lysed on ice for 10 minutes in RIPA lysis buffer (0.5% Na-deoxycholate, 0.1% sodium dodecyl sulfate (SDS), and 1% Nonidet P-40 in phosphate buffered saline (PBS) (Sigma, St. Louis, Missouri) containing protease inhibitor (Roche Diagnostics, Basel, Switzerland). The cell lysates were passed through a 21-gauge needle several times and clarified through centrifugation at 8000 g for 30 minutes at 4°C. The supernatants were collected as the total cell lysates and precipitated with a specific antibody and protein A Sepharose (GE healthcare, Uppsala, Sweden), washed four times with PBST, and dissolved in 2X sample buffer. The immunoprecipitated proteins were separated through 8% SDS-PAGE and transferred to polyvinylidene membranes (Millipore, Billerica, Massachusetts) for immunoblotting with the primary antibodies (diluted in 5% non-fat milk) overnight at 4°C. The secondary antibodies and horseradish peroxidase-conjugated donkey anti-mouse IgG (Santa Cruz) were diluted 1∶5,000 in 5% non-fat milk. The membranes were washed three times prior to detection using the Enhanced Chemiluminescence Kit (Amersham Pharmacia Biotech). Mouse monoclonal anti-Flag and anti-actin antibodies were purchased from Sigma. The anti-hemagglutinin (HA) monoclonal antibody was purchased from Covance Research Products (Emeryville, California). The rabbit polyclonal anti-KIF14 antibody was purchased from Bethyl Laboratories.

### Immunofluorescence Staining

Immunofluorescence staining was performed as previously described [63]. The transfected cells were fixed for 10 minutes at room temperature in 3.7% cold paraformaldehyde in PBS and permeabilized for 10 minutes at room temperature with PBS containing 0.1% Triton X-100. The cells were blocked with PBS containing 3% bovine serum albumin and stained overnight at 4°C with monoclonal anti-HA antibodies, followed by incubation for 1 hour at 37°C with rhodamine-conjugated secondary antibodies (Molecular Probes). They were then mounted with ProLong Gold antifade reagent containing DAPI (Molecular Probes, Eugene, Oregon). The cells were examined and photographed using an LSM 700 META laser-scanning microscope (Carl Ziess MicroImaging Inc.).

### Statistical Analysis

The data were expressed as the means ± standard deviation. The quantitative *in* *vitro* and *in* *vivo* data were analyzed using Student’s *t*-test or one-way ANOVA. A multivariate Cox proportional hazards model was used to investigate the joint association of several risk factors with cancer mortality. For the survival analysis, the log rank test was used to compare the Kaplan-Meier curves of two groups. All statistical tests were two-sided, and P values of less than 0.05 were considered statistically significant.

## Supporting Information

Figure S1 **Anti-KIF14 antibody characterization.** (A) HEK293T cells were transfected with the indicated plasmids. The lysates were used for immunoblotting with KIF14 polyclonal antibodies and KIF14 antibodies pre-absorbed with full-length Flag-KIF14 proteins. The pre-absorption could block the staining in immunoblotting. (B) Characterization of the specificity of the anti-KIF14 polyclonal antibodies used in immunohistochemistry. The tumor tissue specimens from a patient positive for KIF14 expression were stained with KIF14 polyclonal antibodies and KIF14 antibodies pre-absorbed with full-length Flag-KIF14 proteins. Scale bars, 100 µm.
(TIFF)

Figure S2 **The endogenous KIF14 protein levels in lung adenocarcinoma cell lines.** (A) The endogenous KIF14 protein levels in CL cell lines. Left: The CL1-0 and CL1-5 cell lysates were analyzed through immunoblotting using KIF14 antibodies. Actin was used as an internal control. Right: The invasion of CL1-0 and CL1-5 cells was measured using a modified Boyden chambers assay. The invading cells were indicated with propidium iodide staining and quantified (n = 3). (B) The endogenous KIF14 protein levels in lung adenocarcinoma cell lines. The cell lysates were analyzed through immunoblotting using KIF14 antibodies. Actin was used as an internal control.
(TIFF)

Figure S3 **KIF14 expression and cell proliferation in different cell lines.** (A) A cell line transiently expressing KIF14 was established through lentiviral infection into A549 cells, and KIF14 protein expression was assessed through Western blotting with anti-KIF14 antibodies; actin was used as an internal control (left). The cell number was calculated at the indicated times after planting (right). No significant differences were observed in the proliferation rates between the control and KIF14-overexpressing cell lines using one-way ANOVA. The error bars represent the standard deviation of the means. (B) KIF14 expression was knocked down in H1299 cells using shRNA lentiviral infection. After selection with puromycin for two weeks, the KIF14 protein expression patterns were assessed through immunoblotting with anti-KIF14 antibodies; actin was used as an internal control (left). The cell proliferation was calculated at the indicated times after planting (right). The error bars represent the standard deviation of the means.
(TIFF)

Figure S4 **The predicted functional partners of the KIF14 protein.** The list is modified from STRING 9.0 and indicates the calculated scores and published references.
(TIFF)

Figure S5 **KIF14 modulated the distribution of the endogenous CDH11.** CL1-5/vector, CL1-5/KIF14#2, CL1-0/shLacZ and CL1-0/shKIF14 cells were cultured and the membrane fraction was isolated. The protein in the membrane fraction and total cell lysate was analyzed through immunoblotting. The amounts of endogenous CDH11 on membrane fraction were quantified through normalization with the amount in total cell lysates. Hsp90 was used as a cytosol marker.
(TIFF)

Table S1 **Characteristics of 53 lung adenocarcinoma patients determined using real-time quantitative RT-PCR analysis^1^.**
(TIFF)

Table S2 **Hazard ratios for death (from any cause) among patients with lung adenocarcinoma determined using real-time quantitative RT-PCR analysis, according to multivariate Cox regression analysis^1^.**
(TIFF)
