# Supplementary figures and images for: The Motor Protein KIF14 Inhibits Tumor Growth and Cancer Metastasis in Lung Adenocarcinoma
Source: PLoS One. 2013 Apr 23;8(4):e61664. doi: 10.1371/journal.pone.0061664 (PMC3633961; doi:10.1371/journal.pone.0061664)

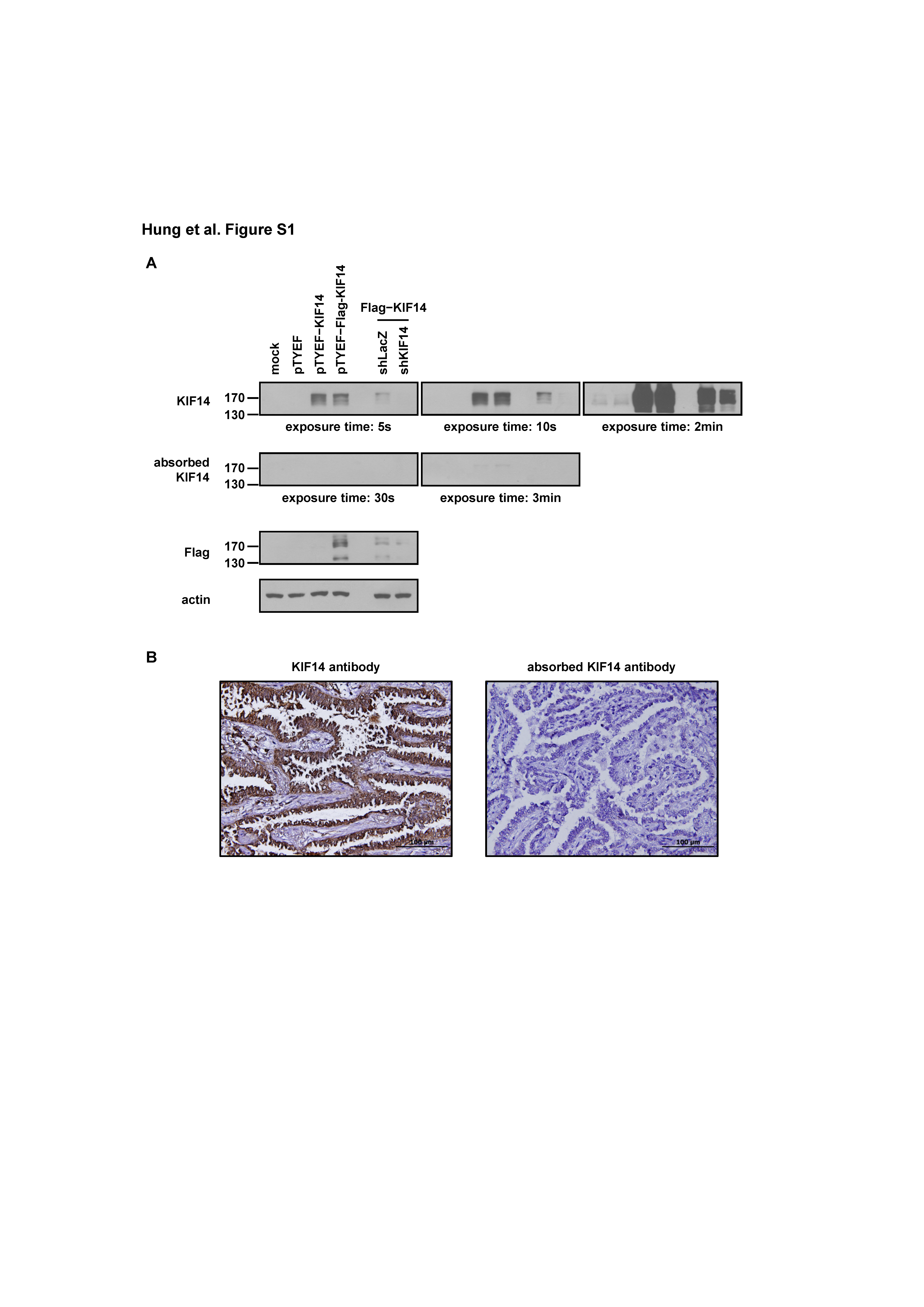

Supplement: Figure S1 — Anti-KIF14 antibody characterization. (A) HEK293T cells were transfected with the indicated plasmids. The lysates were used for immunoblotting with KIF14 polyclonal antibodies and KIF14 antibodies pre-absorbed with full-length Flag-KIF14 proteins. The pre-absorption could block the staining in immunoblotting. (B) Characterization of the specificity of the anti-KIF14 polyclonal antibodies used in immunohistochemistry. The tumor tissue specimens from a patient positive for KIF14 expression were stained with KIF14 polyclonal antibodies and KIF14 antibodies pre-absorbed with full-length Flag-KIF14 proteins. Scale bars, 100 µm. (TIFF) [file pone.0061664.s001.tiff]

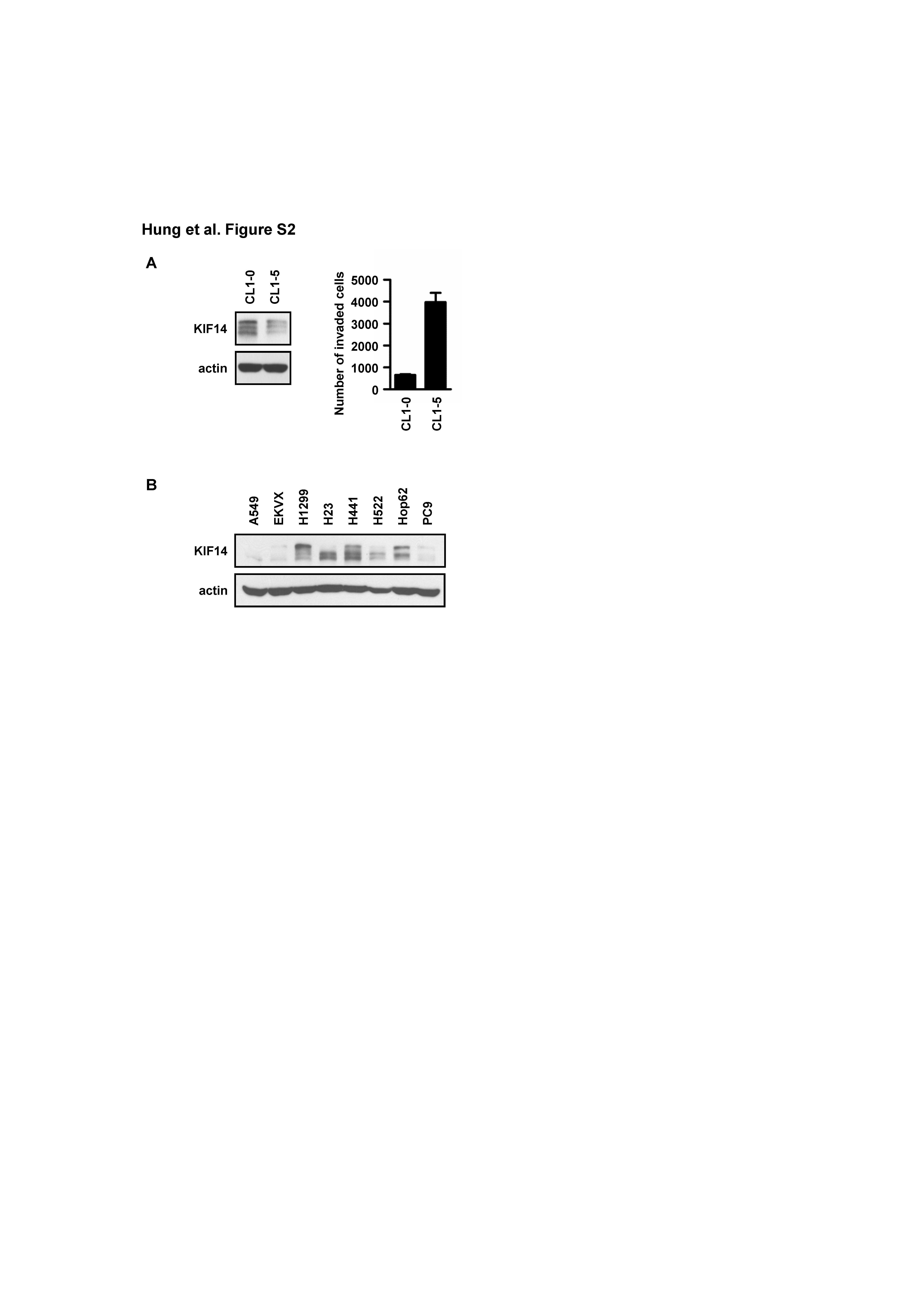

Supplement: Figure S2 — The endogenous KIF14 protein levels in lung adenocarcinoma cell lines. (A) The endogenous KIF14 protein levels in CL cell lines. Left: The CL1-0 and CL1-5 cell lysates were analyzed through immunoblotting using KIF14 antibodies. Actin was used as an internal control. Right: The invasion of CL1-0 and CL1-5 cells was measured using a modified Boyden chambers assay. The invading cells were indicated with propidium iodide staining and quantified (n = 3). (B) The endogenous KIF14 protein levels in lung adenocarcinoma cell lines. The cell lysates were analyzed through immunoblotting using KIF14 antibodies. Actin was used as an internal control. (TIFF) [file pone.0061664.s002.tiff]

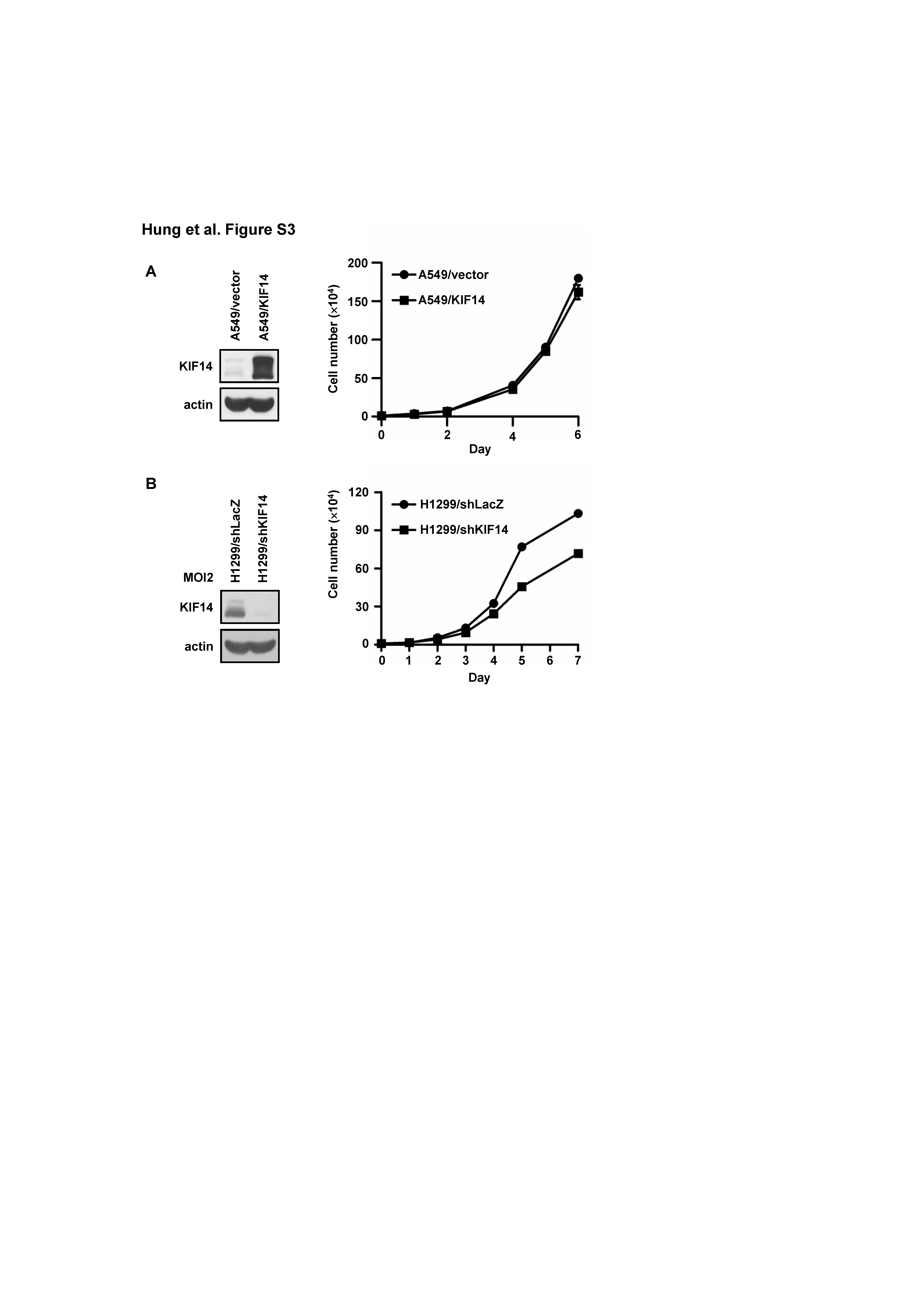

Supplement: Figure S3 — KIF14 expression and cell proliferation in different cell lines. (A) A cell line transiently expressing KIF14 was established through lentiviral infection into A549 cells, and KIF14 protein expression was assessed through Western blotting with anti-KIF14 antibodies; actin was used as an internal control (left). The cell number was calculated at the indicated times after planting (right). No significant differences were observed in the proliferation rates between the control and KIF14-overexpressing cell lines using one-way ANOVA. The error bars represent the standard deviation of the means. (B) KIF14 expression was knocked down in H1299 cells using shRNA lentiviral infection. After selection with puromycin for two weeks, the KIF14 protein expression patterns were assessed through immunoblotting with anti-KIF14 antibodies; actin was used as an internal control (left). The cell proliferation was calculated at the indicated times after planting (right). The error bars represent the standard deviation of the means. (TIFF) [file pone.0061664.s003.tiff]

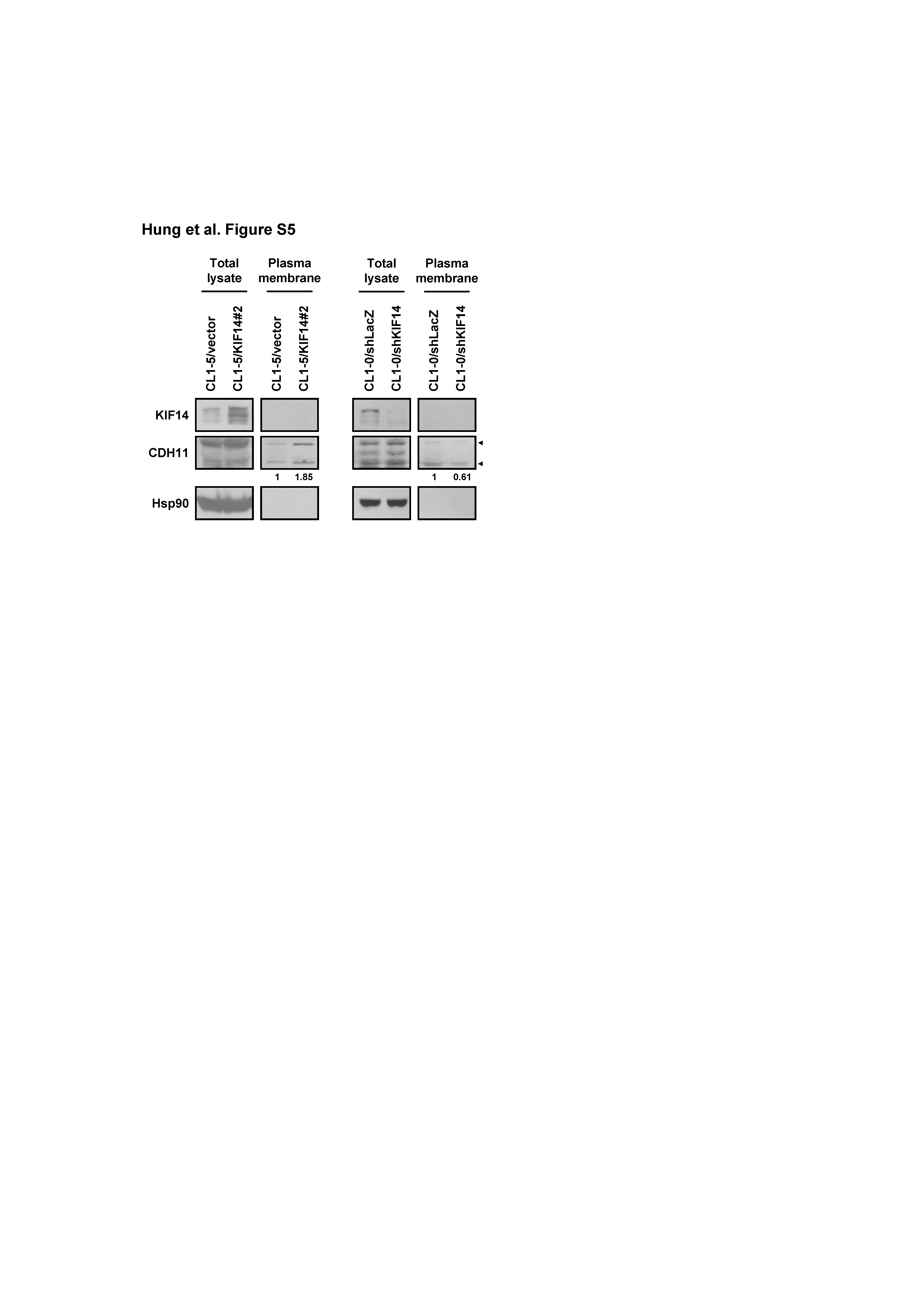

Supplement: Figure S5 — KIF14 modulated the distribution of the endogenous CDH11. CL1-5/vector, CL1-5/KIF14#2, CL1-0/shLacZ and CL1-0/shKIF14 cells were cultured and the membrane fraction was isolated. The protein in the membrane fraction and total cell lysate was analyzed through immunoblotting. The amounts of endogenous CDH11 on membrane fraction were quantified through normalization with the amount in total cell lysates. Hsp90 was used as a cytosol marker. (TIFF) [file pone.0061664.s005.tiff]
